# Microbial Community Dynamics and the Correlation between Specific Bacterial Strains and Higher Alcohols Production in Tartary Buckwheat Huangjiu Fermentation

**DOI:** 10.3390/foods12142664

**Published:** 2023-07-11

**Authors:** Sheng Yin, Mingquan Huang, Jiaxuan Wang, Bo Liu, Qing Ren

**Affiliations:** 1 Key Laboratory of Brewing Molecular Engineering of China Light Industry, Beijing Technology & Business University, Beijing 100048, China; yinsheng@btbu.edu.cn (S.Y.);; 2 Beijing Advanced Innovation Center for Food Nutrition and Human Health, Beijing Technology & Business University, Beijing 100048, China

**Keywords:** Huangjiu, Tartary buckwheat, microbial community dynamics, higher alcohols

## Abstract

Tartary buckwheat is a healthy grain rich in nutrients and medicinal ingredients and consequently is commonly used for Huangjiu brewing. In order to reveal the correlation between microbial succession and higher alcohols production, in this study, Huangjiu fermentation was conducted using Tartary buckwheat as the raw material and wheat Qu as the starter culture. Microbial community dynamics analysis indicated that the bacterial diversity initially decreased rapidly to a lower level and then increased and maintained at a higher level during fermentation. *Lactococcus* was the dominant bacteria and *Ralstonia*, *Acinetobacter*, *Cyanobacteria*, and *Oxalobacteraceae* were the bacterial genera with higher abundances. In sharp contrast, only 13 fungal genera were detected during fermentation, and *Saccharomyces* showed the dominant abundance. Moreover, 18 higher alcohol compounds were detected by GC-MS during fermentation. Four compounds (2-phenylethanol, isopentanol, 1-hexadecanol, and 2-phenoxyethanol) were stably detected with high concentrations during fermentation. The compound 2-ethyl-2-methyl-tridecanol was detected to be of the highest concentration in the later period of fermentation. Correlation analysis revealed that the generation of 2-phenylethanol, isopentanol, 1-hexadecanol, and 2-phenoxyethanol were positively correlated with *Granulicatella* and *Pelomonas*, *Bacteroides*, *Pseudonocardia* and *Pedomicrobium*, and *Corynebacterium*, respectively. The verification fermentation experiments indicated that the improved wheat Qu QT3 and QT4 inoculated with *Granulicatella* T3 and *Acidothermus* T4 led to significant increases in the contents of 2-phenylethanol and pentanol, as well as isobutanol and isopentanol, respectively, in the Tartary buckwheat Huangjiu. The findings benefit understanding of higher alcohols production and flavor formation mechanisms in Huangjiu fermentation.

## 1. Introduction

Huangjiu (Chinese rice wine) is one of the most famous traditional fermented liquors in China and has a brewing history of thousands of years [1]. Generally, Huangjiu brewing uses various grains (rice or millet) as raw materials and the wheat Qu (solid compounds consisting of wheat, various microorganisms, and complex enzyme systems) as the fermentation starter culture, which contribute to the unique organoleptic qualities and characteristics [2]. Huangjiu is popular due to not only its desirable flavor but also rich nutrition and health-promoting ingredients, including polyphenols, polysaccharides, oligosaccharides, peptides, organic acids, and trace elements [3]. Tartary buckwheat is regarded as a healthy grain rich in nutrients and medicinal ingredients and could be of benefit in the prevention of cardiovascular and cerebrovascular diseases [4]. Hence, Tartary buckwheat is commonly used as the raw material for healthcare Huangjiu brewing.

The characteristics of Huangjiu are defined by complex flavor compounds, including acids, alcohols, esters, and aldehydes [5], and the style, flavor, and taste of Huangjiu is influenced by different raw materials, starter cultures, production regions, and brewing processes. Those volatile compounds are generated from the fermentation of raw materials with the involvement of the metabolic activity of the microbial community and enzymes [5]. Higher alcohols are essential volatile flavor compounds of fermented liquors [6]. The proportions and relative levels of higher alcohols significantly affect the sensory quality and safety of alcoholic beverage products. The appropriate content and proportion of higher alcohols impart a characteristic flavor of mellowness, mildness, tenderness, and harmony [7], while they generate an unpleasant taste and strong intoxicating feeling when the contents are above a certain threshold [8]. Furthermore, higher alcohols are the substrates for acetate esters biosynthesis [9], which are another important group of volatile flavor compounds in alcoholic beverages. Consequently, the production and control of higher alcohols during alcoholic beverage fermentation has attracted sustained attention.

The *Saccharomyces* yeasts are generally recognized as the major contributors of higher alcohols in alcoholic fermentation, which could be biosynthesized from branched-chain and aromatic amino acid catabolism through the Ehrlich pathway [10,11]. Various factors could influence the compositions of higher alcohols produced by yeasts, such as glucose metabolism, specific branched-chain amino acid concentrations and compositions in fermentation media, and process parameters, including oxygen availability and temperature [12,13]. 2-methyl-1-propanol and 2-phenylethanol are regarded as the important flavor compounds of Chinese Huangjiu, and they have relatively high concentrations at the end of fermentation [14]. Isobutanol, isoamyl alcohol, and phenylethyl are the major higher alcohols of most important flavor components in fresh Shaoxing Huangjiu [15,16], and positive correlations have been found between phenylethanol production and *Saccharomycopsis* and between isopentanol production and *Weissella* in Shaoxing Huangjiu fermentation [17]. During black glutinous rice Huangjiu fermentation, *Candida* and *Monascus* are involved in phenylethanol production derived from phenylalanine metabolism [14]. The compounds 2-methyl-1-propanol, 3-methyl-1-butanol, 2,3-butanediol, and 2-phenylethanol have been detected during sorghum Huangjiu fermentation, and the production of 2-methyl-1-propanol is correlated with *Ralstonia* [18]. However, in the complex fermentation system of Huangjiu, the roles of other essential non-*Saccharomyces* microbes in higher alcohols production remain poorly understood. Undoubtedly, it is indispensable to reveal the causal relationship between higher alcohols production and the interaction of diverse microbes and raw materials for a comprehensive understanding of flavor formation during Chinese traditional Huangjiu fermentation.

In this study, the dynamic changes in microbial community and higher alcohols production during Tartary buckwheat Huangjiu fermentation were monitored, and a correlation analysis of different microbial genera and higher alcohol compounds was performed. These results are of benefit to generating a comprehensive understanding of the mechanism of higher alcohols biosynthesis during Huangjiu fermentation and provide a possible strategy for higher alcohols control in Huangjiu products.

## 2. Materials and Methods

### 2.1. Tartary Buckwheat Huangjiu Fermentation and Samples Preparation

One kilogram of Tartary buckwheat (Zhangjiakou, Hebei, China) was washed and soaked in clean water for 24 h and then cooked in clean water (three times the volume) for 1 h. Glucoamylase (0.15%, *w*/*w*) was added to the well-cooked Tartary buckwheat for saccharification for 60 min. The wheat Qu (15%, *w*/*w*, BeiZong Huangjiu Company, Zhangjiakou, Hebei, China) and yeast (0.15%, *w*/*w*) were added to the saccharified mixture and mixed thoroughly for fermentation. Primary fermentation was performed in a tank at 30 °C for 10 days, and secondary fermentation continued at 15 °C for another 30 days. The fermentation samples were prepared every two days for higher alcohols determination and microbial DNA extraction.

### 2.2. Microbial Community Analysis during Tartary Buckwheat Huangjiu Fermentation

#### 2.2.1. DNA Isolation and Illumina MiSeq Sequencing

Metagenomic DNA was extracted from the fermentation samples using E.Z.N.A. soil DNA kit (Omega Bio-tek, Norcross, GA, USA). DNA quantification was conducted using NanoDrop 2000 Spectrophotometer (Thermo Scientific, Wilmington, NC, USA). The 16S rDNA and ITS1 amplicons were generated by PCR from 50 ng of genomic DNA in GeneAmp 9700 System (ABI, Los Angeles, CA, USA), respectively. The V3-V4 hypervariable regions of bacterial 16S rDNA were amplified using the forward primer F338 (5′-ACTCCTACGGGAGGCAGCAG-3′) and the reverse primer R806 (5′-GGACTACHVGGGTWTCTAAT-3′) [19]. The PCR reaction was performed with the following cycling conditions: 95 °C for 5 min; 25 cycles of 95 °C for 30 s, 55 °C for 30 s, and 72 °C for 40 s; and 72 °C for 10 min. In addition, the hypervariable ITS region of relatively conserved fungi rRNA was amplified using the forward primer ITSF (5′-CTTGGTCATTTAGAGGAAGT-3′) and the reverse primer ITSR (5′-GCTGCGTTCTTCATCGATGC-3′) [20]. The PCR reaction was performed with the following cycling conditions: 95 °C for 5 min; 30 cycles of 95 °C for 30 s, 61 °C for 30 s, and 72 °C for 45 s; and 72 °C for 10 min. The purified amplicons in equal molar ratios were loaded on Illumina MiSeq platform (Illumina, San Diego, CA, USA). A 2 × 300 paired-end configuration was conducted for sequencing. Image analysis and base calling were performed using the MiSeq Control Software 3.1.

#### 2.2.2. Sequencing Data Analysis

The sequences were filtered by Trimmomatic for analysis. The effective sequences preclustered at a 97% identity were classified into operational taxonomic units (OTUs) by Usearch (http://drive5.com/uparse/). The OTUs of 16S rRNA were assigned taxonomic categories using the ribosomal database program (RDP) classifier at a confidence threshold of 70% and then predicted to the species level using the Silva 16S rRNA database. The OTUs of ITS were analyzed using the Unite program (http://unite.ut.ee/index.php). The calculations of the alpha and beta diversity statistics were conducted after sequence rarefication. Microbial community diversity analysis was performed by the alpha diversity using the Simpson index. Species correlation was calculated using the Pearson correlation coefficient.

### 2.3. Higher Alcohols Determination during Tartary Buckwheat Huangjiu Fermentation

Higher alcohols produced during the Tartary buckwheat Huangjiu fermentation were determined by headspace solid phase microextraction (HS-SPME) sampling combined with gas chromatography and mass spectrometry (GC-MS) analysis. The fermentation sample was centrifuged at 8000× *g* at 4 °C for 10 min, and the supernatant was collected and filtered through a 0.22 μm filter. The filtered supernatant (0.5 mL), 15% ethanol (5.44 mL), and the internal standard substance 2-octanol (8.8 mg/L, 60 μL) were added in a 15 mL headspace vial. Analytes were extracted by SPME for 45 min in water bath (50 °C) with ultrasonication using an adapter equipped with a 50/30 μm DVB/CAR/PDMS fiber (1 cm, Supelco Inc., Bellefonte, PA, USA). The fiber was then inserted into the injection port of the GC for thermal desorption at 250 °C for 7 min.

GC-MS analysis was conducted on Shimadzu GC-MS 2010Plus system (Shimadzu, Kyoto, Japan) with a DB column (30 m × 0.25 mm i.d., 0.25 μm film thickness). Helium (99.999 purity) was used as the column carrier gas at a constant flow rate of 1.0 mL/min. The injector and detector temperature was set at 250 °C. The oven temperature was held at 40 °C for 3 min, raised to 100 °C at a rate of 6 °C/min, and then raised to 230 °C at a rate of 10 °C/min and was held at 230 °C for 7 min. The MS transfer line temperature and ion source temperature were set at 230 °C. Electron ionization (EI, 50 μA electric current, 70 eV electron) was used as the ionization mode for GC-MS analysis. The ion currents within the mass range from 33 to 400 were monitored for analysis.

The content of higher alcohols was calculated by the internal standard substance 2-octanol detected by GC-MS according to the following formula.

$$C=12\frac{Ac}{Ais}Cis$$

where C is the content of higher alcohol, μg/L; *Cis* is the content of 2-octanol, μg/L; *Ac* is the peak area of higher alcohol; and *Ais* is the peak area of 2-octanol.

### 2.4. Correlation Analysis between Microbes and High Alcohols Production during Tartary Buckwheat Huangjiu Fermentation

Significant correlations between microbes at the genus level and higher alcohols production were established by the Pearson correlation coefficient. The Benjamini–Hochberg procedure was used to adjust *p*-values (*p* ≤ 0.05). The correlation network was built using R Programming.

### 2.5. Verification of the Correlation between the Wheat Qu Bacterial Strains and High Alcohols Production during Tartary Buckwheat Huangjiu Fermentation

In order to verify the correlation between specific microbial strains and high alcohols production, four bacterial strains (*Lactococcus* T1, *Ralstonia* T2, *Granulicatella* T3, and *Acidothermus* T4) isolated from the wheat Qu were used for the improvement of wheat Qu making. Briefly, the smashed wheat was mixed with clean water (20%, *w*/*w*) and the fresh cultures (1%, *v*/*v*) of different bacterial strains T1, T2, T3, and T4, respectively; the mixture was stirred thoroughly and compacted into piles, and spontaneous fermentation was performed at 40~45 °C for 30 days. The original wheat Qu (Q) and the improved wheat Qu added with bacterial strains (QT1, QT2, QT3, and QT4) were used for the Tartary buckwheat Huangjiu fermentation as previously described, respectively. Higher alcohols production during Huangjiu fermentation was determined, as previously described.

## 3. Results

### 3.1. Dynamic Change in Bacterial Community during Tartary Buckwheat Huangjiu Fermentation

**Figure 1:**
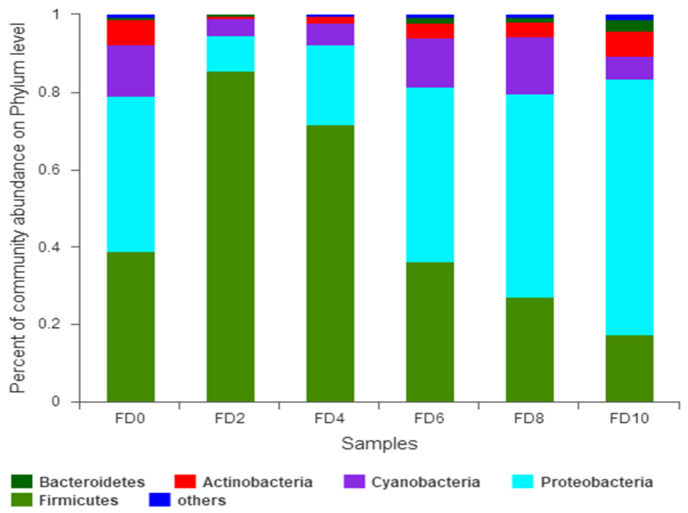
Relative abundance of bacterial phyla during Tartary buckwheat Huangjiu fermentation. FD, fermentation day.

**Figure 2:**
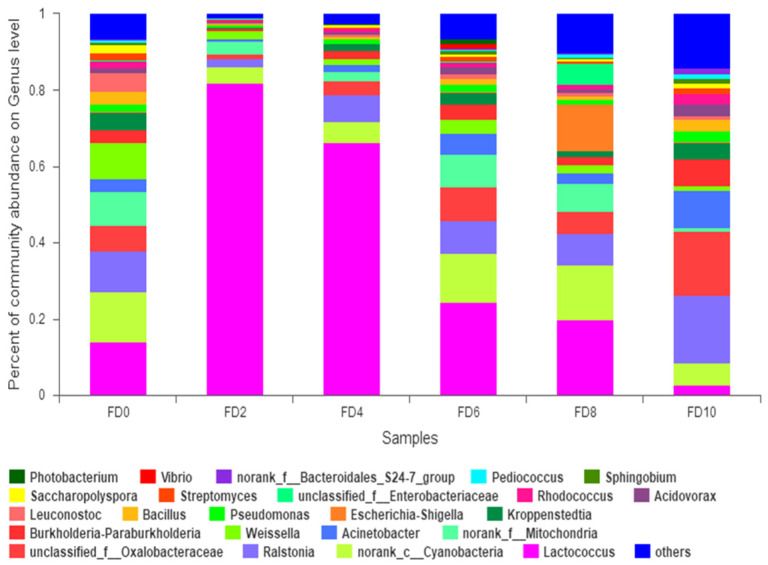
Relative abundance of bacterial genera during Tartary buckwheat Huangjiu fermentation. FD, fermentation day.

**Figure 3:**
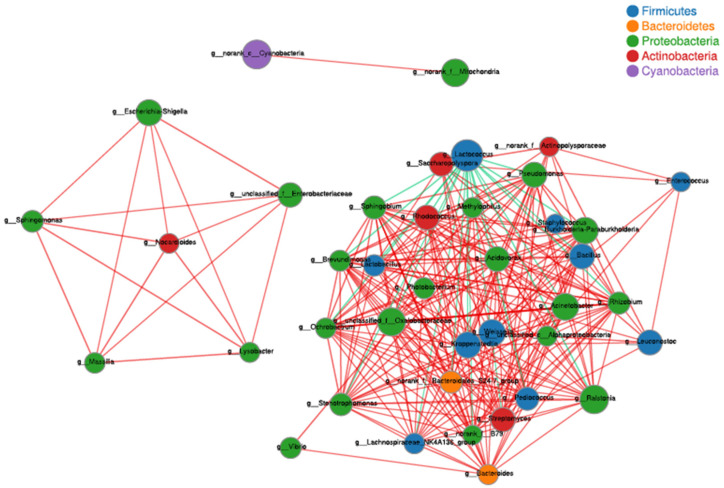
Correlation network of bacterial genera in samples of Tartary buckwheat Huangjiu fermentation. The red and blue lines refer to positive correlations (*p* ≤ 0.05) and negative correlations (*p* ≤ 0.05).

**Figure 4:**
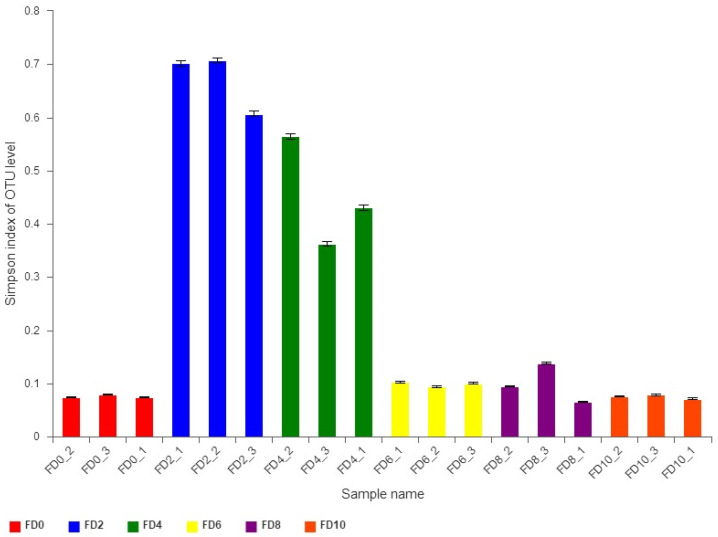
Alpha diversity analysis of bacteria in samples of Tartary buckwheat Huangjiu fermentation. FD, fermentation day.

**Figure 5:**
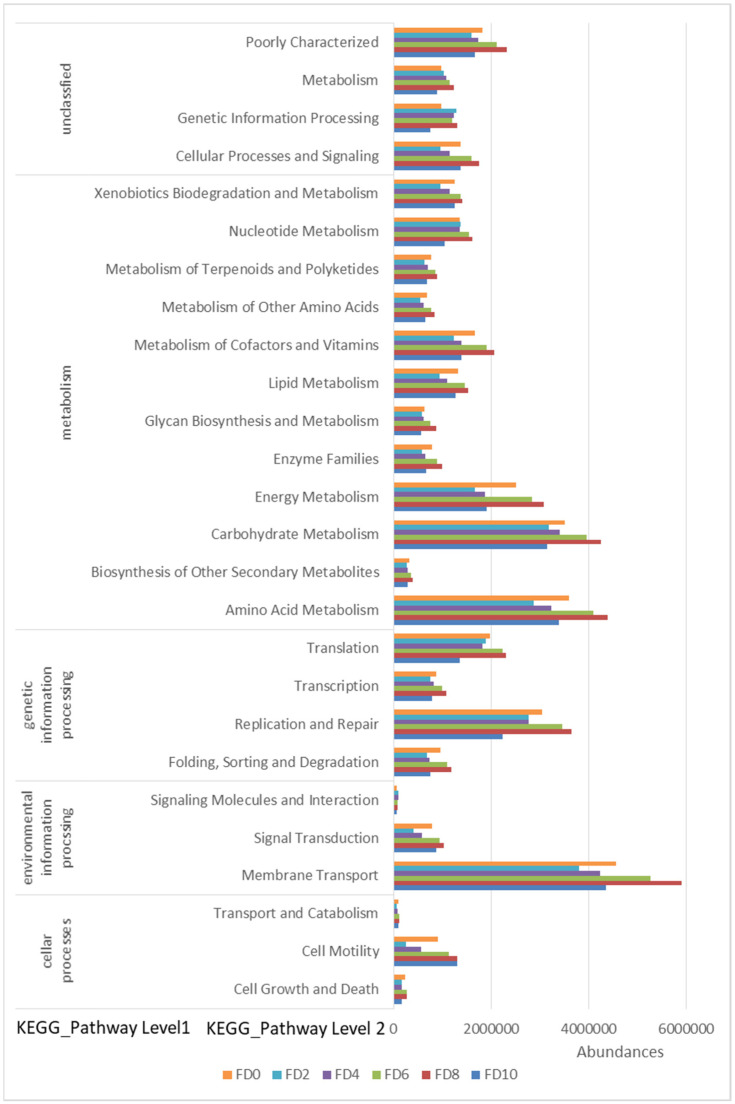
Relative abundance levels of predicted gene functions related to KEGG pathways in bacteria during Tartary buckwheat Huangjiu fermentation. FD, fermentation day.

To reveal the dynamic change in the bacterial community during the Tartary buckwheat Huangjiu fermentation, the microbial DNA from different fermentation samples was subject to high-throughput sequencing and analysis. The data showed that 981 bacterial OTUs were classified from 1,002,498 qualified sequencing fragments in all fermentation samples. Sequence analysis indicated that those bacterial OTUs could be assigned to 30 phyla, 66 classes, 133 orders, 232 families, and 482 genera. As shown in Figure 1, *Firmicutes* and *Proteobacteria* had the highest abundance at the level of phylum during the Tartary buckwheat Huangjiu fermentation. *Firmicutes* dominated at the beginning of fermentation, where their percentage reached 85.52% on day two, and then they decreased gradually. *Proteobacteria* showed an opposite dynamic trend in comparison with *Firmicutes.* In addition, the abundance of *Actinobacteria* and *Bacteroidetes* was relatively high. At the genus level, *Lactococcus* was the dominant microbe, which rapidly increased to a peak percentage of 80% on day two and then decreased gradually during fermentation (Figure 2). *Ralstonia*, *Acinetobacter*, *Cyanobacteria*, and *Oxalobacteraceae* also had a higher abundance. Species correlation analysis (Figure 3) indicated that *Lactococcus* was negatively correlated with 20 bacterial genera, such as *Lactobacillus* and *Sphingobium*. There was a positive correlation among *Escheria-shigella*, *Sphingomonas*, *Massilia*, *Lysobacter*, *Nocardioides*, and *Enterobacteriaceae*. In the correlation network, *Rhizobium*, *Ochrobactrum*, and *Streptomyces* played an important role as the connection hub. Alpha diversity analysis showed that the Simpson index reached higher values on day two and four of fermentation, revealing that the bacterial diversity first rapidly decreased to a lower level and then increased and maintained at a higher level during fermentation (Figure 4). The gene functions prediction related to KEGG pathways based on sequencing data analysis revealed that different samples exhibited similar gene functions during fermentation. But large differences were observed in the relative abundance of gene functions from different samples. As shown in Figure 5, the main metabolic pathways of bacteria during fermentation were mainly enriched in membrane transport, amino acid metabolism, and carbohydrate metabolism. The abundances in carbohydrate metabolism and amino acid metabolism gradually increased at first and then decreased at the end of fermentation in 10 days, which was in accordance with the dynamic change in higher alcohols generation. This revealed that bacteria utilized carbohydrates from Tartary buckwheat and proliferated quickly along with the fermentation and simultaneously produced higher alcohols from amino acid metabolism via the Ehrlich pathway.

### 3.2. Dynamic Change in Fungal Community during Tartary Buckwheat Huangjiu Fermentation

**Figure 6:**
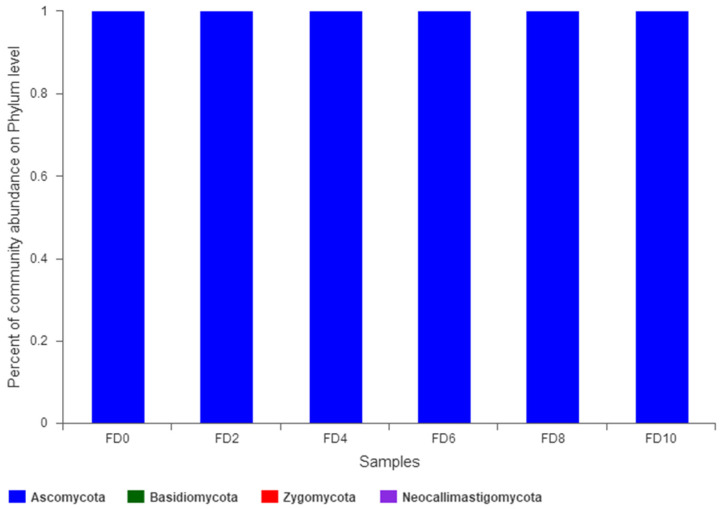
Relative abundance of fungal phyla during Tartary buckwheat Huangjiu fermentation. FD, fermentation day.

**Figure 7:**
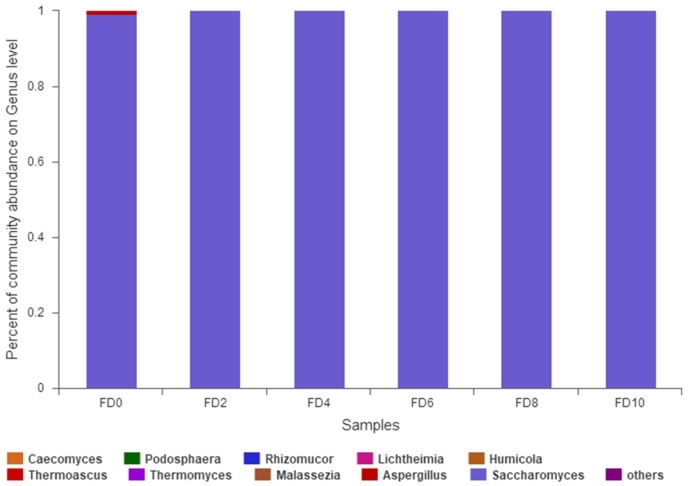
Relative abundance of fungal genera during Tartary buckwheat Huangjiu fermentation. FD, fermentation day.

**Figure 8:**
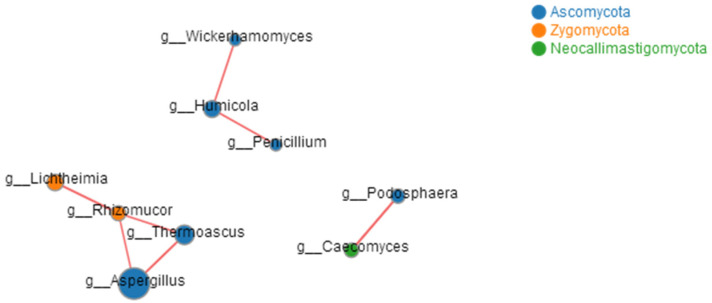
Correlation network of fungal genera in samples of Tartary buckwheat Huangjiu fermentation. The red lines refer to positive correlations (*p* ≤ 0.05).

**Figure 9:**
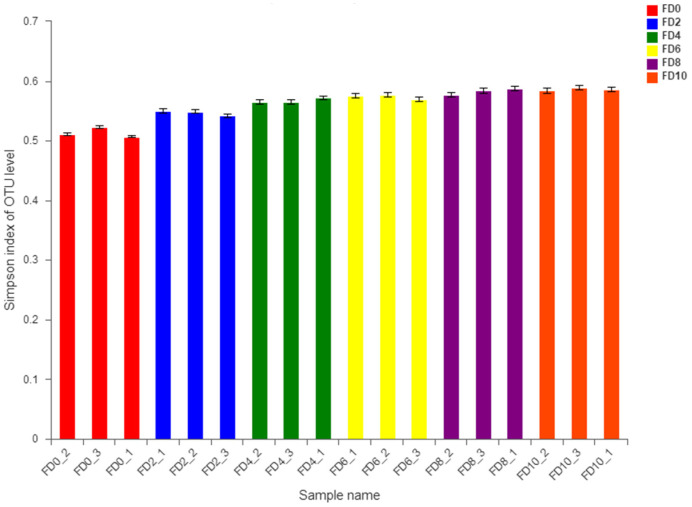
Alpha diversity analysis of fungi in samples of Tartary buckwheat Huangjiu fermentation. FD, fermentation day.

In sharp contrast to the complex bacterial community, only 20 fungal OTUs were classified from 659,656 qualified sequencing fragments in all the fermentation samples. Those OTUs could be assigned to 1 kingdom, 4 phyla, 7 classes, 7 orders, 11 families, and 13 genera. At the phylum level, *Ascomycota* absolutely dominated in abundance in all the fermentation samples (Figure 6). At the genus level, *Saccharomyces* showed absolute dominance in abundance in all the fermentation samples (Figure 7), but *Aspergillus* and *Malassezia* with extremely low abundance were detected at the beginning of fermentation. Correlation analysis (Figure 8) indicated that *Wickerhamomyces* played an important role as the connection hub in the correlation network, which was positively correlated with *Penicillium* and *Humicola*. *Rhizomucor* was found to be located near the center of the correlation network and might play a key role in maintaining the tight connection of the whole network. A mutual positive correlation existed among *Rhizomucor*, *Thermoascus*, and *Aspergillus*. Alpha diversity analysis showed that the Simpson index increased slightly during fermentation, revealing that the fungal diversity gradually decreased. However, no significant difference in the fungal diversity was observed in different fermentation samples (Figure 9).

### 3.3. Higher Alcohols Production during Tartary Buckwheat Huangjiu Fermentation

Eighteen higher alcohol compounds were detected by GC-MS during the 10 days of the Tartary buckwheat Huangjiu fermentation, as shown in Table 1. Most higher alcohol compounds appeared in the early four days of fermentation, and then several compounds dominated till the end of fermentation. Generally, four compounds (2-phenylethanol, isopentanol, 1-hexadecanol, and 2-phenoxyethanol) were stably detected with high concentrations in samples of different fermentation stages, which might significantly affect the flavor of Huangjiu. In addition, abundant 2-ethyl-2-methyl-tridecanol was detected during the later period of fermentation, whose concentration was the highest among all the higher alcohols. Pentanol with a quite high concentration was only detected on the eighth day of fermentation.

### 3.4. Correlation Analysis of Microbes and Higher Alcohols Production during Tartary Buckwheat Huangjiu Fermentation

The production of 18 higher alcohol compounds was correlated with 294 microbial genera (*p* ≤ 0.05). The generation of 2-phenylethanol was positively correlated with seven genera, including *Granulicatella*, *Pelomonas*, *Coprococcus*, *Oscillibacter*, *Ruminiclostridium*, *Ruminococcaceae*, and *Deltaproteobacteria*. The production of pentanol was positively correlated with 72 genera, such as *Rhodopseudomonas*, while isopentanol was positively correlated with 31 genera, such as *Bacteroides*. Though *Clostridium* is the natural producer of butanol, *Clostridium sensu stricto* and 51 other genera, such as *Acidothermus*, were found to be positively correlated with isobutanol generation, but a negative correlation was observed between *Ignavibacterium* and isobutanol production. The appearance of 2-phenoxyethanol was positively correlated with 79 genera, such as *Corynebacterium*. As for alkanol compounds, 1-hexadecanol was positively correlated with 72 genera, such as *Pseudonocardia* and *Pedomicrobium*, and 1-hendecanol was positively correlated with 72 genera, such as *Agromyces*.

### 3.5. Effects of Improved Wheat Qu Inoculated with Specific Bacterial Strains on Higher Alcohols Production in Tartary Buckwheat Huangjiu Fermentation

The aforementioned results indicated that *Lactococcus* and *Ralstonia* were the dominant bacterial genera during Huangjiu fermentation, and *Granulicatella* and *Acidothermus* were positively correlated with the production of 2-phenylethanol and isobutanol. In order to verify the roles of specific bacterial strains in higher alcohols production, the bacterial strains *Lactococcus* T1, *Ralstonia* T2, *Granulicatella* T3, and *Acidothermus* T4 isolated from the wheat Qu were used for the improved wheat Qu making and consequent Huangjiu fermentation. The fermentation results (Table 2) showed that the improved wheat Qu QT1 and QT2 inoculated with *Lactococcus* T1 and *Ralstonia* T2 hardly led to significant differences in higher alcohols production. In sharp contrast, the improved wheat Qu QT3 and QT4 inoculated with *Granulicatella* T3 and *Acidothermus* T4 indeed resulted in significant increases in the contents of several specific higher alcohol compounds and total higher alcohols in Huangjiu. The additional inoculation of *Granulicatella* T3 in QT3 contributed to the higher contents of 2-phenylethanol and pentanol, and *Acidothermus* T4 in QT4 gave rise to more isobutanol and isopentanol generation. These results verified the positive correlations between *Granulicatella* and 2-phenylethanol production and *Acidothermus* and isobutanol generation during the Tartary buckwheat Huangjiu fermentation. It also revealed that *Granulicatella* and *Acidothermus* might get involved in the biosynthesis of multiple higher alcohol compounds during Huangjiu fermentation.

## 4. Discussion

In different regions, Chinese Huangjiu has distinguish local flavor characteristics due to the diversities in raw materials, microbial starter cultures (Qu), and brewing techniques [21,22,23]. Therefore, understanding the correlation between flavor characteristics and raw materials and microbial metabolisms is indispensable for the quality control of Huangjiu products. In this study, the featured Huangjiu made from Tartary buckwheat rich in functional components was used for the analysis of microbial community dynamics and higher alcohols production to establish a correlation between flavor formation and raw materials and microbial fermentation.

The microbes in Qu are the core driving force for Huangjiu brewing and they play an essential role in flavor compounds generation [23,24,25]. Previous studies showed that significant variances in microbial communities were observed in different Huangjiu Qu samples, and the dominant microbes with high abundances included *Bacillus*, *Weissella*, *Pediococcus*, *Pantoea*, *Lactobacillus*, *Rhizopus*, and *Saccharomyces* [21,23,24]. However, the dominant microbes during the Tartary buckwheat Huangjiu fermentation were quite different, which included *Lactococcus*, *Ralstonia*, *Acinetobacter*, *Cyanobacteria*, *Oxalobacteraceae*, and *Saccharomyces*. The significant variety evidently reflected the distinctive features of the local wheat Qu and raw materials used for Huangjiu brewing.

The formation of flavor compounds in Huangjiu were closely related with the raw materials and starter cultures used in the fermentation [22,23]. During the Tartary buckwheat Huangjiu brewing, several higher alcohol compounds, including 2-phenylethanol, 2-phenoxyethanol, 2-ethyl-2-methyl-tridecanol, and 1-hexadecanol, were constantly detected from the beginning to the end of the fermentation, and their concentrations remarkably increased along with the fermentation. It revealed that those compounds were generated from both Tartary buckwheat and starter cultures fermentation. The compounds that only appeared on day 0 probably were released from Tartary buckwheat. In addition, some compounds arose along with fermentation and disappeared afterwards, which probably were the intermediate metabolites produced by fermentation microbes. The specific contribution of Tartary buckwheat to the flavor formation of the fermented Huangjiu remains a mystery due to the lengthy fermentation process driven by the wheat Qu and various microbes, as well as the complex chemical components of Tartary buckwheat. Tartary buckwheat contains proteins, polysaccharide, polyphenolic compounds, multiple vitamins, cellulose, weakly alkaline starch, various trace elements, dietary fiber, and various kinds of essential amino acids [26]. These components could be transformed by microbial metabolism or directly released into the fermentation system, exerting positive or negative impacts on the flavor of Huangjiu. But it is a challenge to trace the track of specific components from Tartary buckwheat to fermented Huangjiu.

2-phenylethanol is commonly detected in Huangjiu, and its presence contributes to the favorable rose-like odor [27]. Natural 2-phenylethanol can be extracted from plants or produced by microbial fermentation [28,29]. Various yeasts and fungi possess the capacity of 2-phenylethanol biosynthesis from L-phenylalanine by the sequential catalysis of transaminase, decarboxylase, and alcohol dehydrogenase via the Ehrlich pathway [30,31,32,33,34,35,36,37]. Few prokaryotes have been reported to produce 2-phenylethanol [38]. Some wild bacteria were found to produce 2-phenylethanol via different metabolic pathways. *Enterobacter* sp. CGMCC 5087 was identified to be able to de novo synthesize 2-phenylethanol from glucose through the shikimate/phenylpyruvate pathway [39]. *Proteus mirabilis* JN458 was verified to produce 2-phenylethanol by L-amino acid deaminase, α-keto acid decarboxylase, and alcohol dehydrogenase [40]. In this work, a large amount of 2-phenylethanol was detected during the Tartary buckwheat Huangjiu fermentation, and its generation was positively correlated with seven genera, including *Coprococcus*, *Oscillibacter*, *Ruminiclostridium*, *Ruminococcaceae*, *Deltaproteobacteria*, *Granulicatella*, and *Pelomonas*, which are possible candidates for 2-phenylethanol production.

2-phenoxyethanol was detected with a quite higher concentration during the Tartary buckwheat Huangjiu fermentation. Obviously, this aromatic compound possibly exerts an important effect on the flavor of Huangjiu. The presence of 2-phenoxyethanol was due to both Tartary buckwheat and microbial fermentation. Seventy-nine genera of microbes such as *Corynebacterium* were possible producers of 2-phenoxyethanol. To our knowledge, there is no existing report on 2-phenoxyethanol biosynthesis in microorganisms. As this compound is an important chemical material and mainly produced by chemosynthesis [41], the findings provide possible novel methods for natural 2-phenoxyethanol production by microbial fermentation.

Higher alkanol compounds are commonly rich in plants and possess important biological activities [42]. Three higher alkanol compounds were detected during the Tartary buckwheat Huangjiu fermentation. 1-hexadecanol was detected at the beginning of fermentation, and its final concentration was extremely high by the end. It is reasonable to conclude that Tartary buckwheat provided the source of 1-hexadecanol, and its generation was enhanced by subsequent microbial fermentation. The presence of high concentrations of higher alkanol compounds highlights the healthcare feature of the Tartary buckwheat Huangjiu product.

Four bacterial strains from the dominant genera and the genera correlated with 2-phenylethanol and isobutanol biosynthesis during fermentation were utilized for the improved wheat Qu making and Huangjiu brewing. The validation fermentation experiments confirmed the positive correlation between specific bacterial strains and higher alcohols production. Though more validations are needed, useful cues provided by correlation analysis generate more possibilities for the development of distinguished starter cultures (Qu) to bring about an improvement in the quality of Chinese Huangjiu.

## 5. Conclusions

During the Tartary buckwheat Huangjiu fermentation, *Lactococcus*, *Ralstonia*, *Acinetobacter*, *Cyanobacteria*, *Oxalobacteraceae*, and *Saccharomyces* were the genera with the higher abundances. Eighteen higher alcohol compounds were detected during fermentation, and 2-phenylethanol, isopentanol, 1-hexadecanol, and 2-phenoxyethanol were stably detected with high concentrations. The generation of 2-phenylethanol, isopentanol, 1-hexadecanol, and 2-phenoxyethanol was positively correlated with *Granulicatella* and *Pelomonas*, *Bacteroides*, *Pseudonocardia* and *Pedomicrobium*, and *Corynebacterium*, respectively. The improved wheat Qu QT3 and QT4 inoculated with *Granulicatella* T3 and *Acidothermus* T4 led to significant increases in the contents of 2-phenylethanol and pentanol, and isobutanol and isopentanol, respectively, in the Tartary buckwheat Huangjiu.

## Figures and Tables

**Table 1:** Higher alcohols production (µg/L) during Tartary buckwheat Huangjiu fermentation.

	Day 0	Day 2	Day 4	Day 6	Day 8	Day 10
2-phenylethanol	14.56 ± 1.35	312.76 ± 2.47	811.16 ± 1.14	1551.68 ± 0.82	1728.42 ± 0.91	1596.30 ± 1.00
isobutanol	ND	26.37 ± 1.24	37.42 ± 1.07	317.34 ± 1.10	ND	ND
isopentanol	ND	237.82 ± 1.72	396.22 ± 0.87	2089.44 ± 2.22	ND	2513.07 ± 3.21
pentanol	ND	ND	ND	ND	2102.71 ± 1.41	ND
isooctanol	ND	5.48 ± 2.85	57.53 ± 0.81	ND	ND	ND
methionol	ND	3.91 ± 0.66	10.02 ± 0.55	ND	ND	ND
2-phenoxyethanol	14.54 ± 1.07	15.89 ± 0.56	161.50 ± 1.04	ND	908.45 ± 0.94	1760.51 ± 1.08
2-ethyl-2-methyl-tridecanol	79.56 ± 1.98	ND	ND	24439.67 ± 4.88	9051.63 ± 0.66	ND
1-hendecanol	ND	ND	ND	ND	350.83 ± 23.54	ND
1-dodecanol	40.42 ± 1.58	ND	ND	ND	ND	ND
1-hexadecanol	27.60 ± 0.29	12.57 ± 1.53	62.29 ± 1.19	ND	4343.59 ± 0.41	ND
linalool	ND	ND	46.32 ± 1.05	ND	ND	ND
lavandulol	ND	ND	17.66 ± 1.33	ND	ND	ND
linoleny alcohol	ND	ND	150.80 ± 53.36	ND	ND	ND
2-(2-ethylhexyloxy) ethanol	ND	ND	ND	ND	517.87 ± 0.55	ND
3,3-dimethyl-2-butanol	ND	ND	ND	465.31 ± 1.94	ND	ND
2-(2-butoxyethoxy) ethanol	ND	29.45 ± 1.06	70.19 ± 0.89	ND	ND	ND
2-isopropyl-5-methyl-1-heptanol	13.53 ± 0.54	ND	72.11 ± 1.05	ND	ND	ND

ND, not detected.

**Table 2:** Higher alcohols production (mg/L) in Tartary buckwheat Huangjiu fermented with the improved wheat Qu inoculated with specific bacterial strains.

Higher Alcohols	Qu	QT1	QT2	QT3	QT4
isopentanol	2.65 ± 0.08 ^a^	2.81 ± 0.12 ^a^	2.57 ± 0.10 ^a^	2.53 ± 0.09 ^a^	2.84 ± 0.12 ^b^
2-phenylethanol	1.83 ± 0.11 ^a^	1.77 ± 0.13 ^a^	1.79 ± 0.12 ^a^	2.26 ± 0.14 ^b^	1.81 ± 0.09 ^a^
2-phenoxyethanol	1.76 ± 0.10 ^a^	1.68 ± 0.09 ^a^	1.80 ± 0.13 ^a^	1.83 ± 0.12 ^a^	1.79 ± 0.12 ^a^
pentanol	0.62 ± 0.07 ^a^	0.70 ± 0.05 ^a^	0.57 ± 0.06 ^a^	0.83 ± 0.08 ^b^	0.58 ± 0.06 ^a^
isooctanol	0.04 ± 0.001 ^a^	0.03 ± 0.001 ^a^	0.03 ± 0.001 ^a^	0.03 ± 0.001 ^a^	0.04 ± 0.002 ^a^
isobutanol	0.03 ± 0.002 ^a^	0.04 ± 0.001 ^a^	0.04 ± 0.002 ^a^	0.04 ± 0.001 ^a^	0.72 ± 0.011 ^b^
others	0.81 ± 0.07 ^a^	0.72 ± 0.08 ^a^	0.74 ± 0.05 ^a^	1.25 ± 0.11 ^b^	1.18 ± 0.12 ^b^
total content	7.65 ± 0.14 ^a^	7.75 ± 0.16 ^a^	7.54 ± 0.13 ^a^	8.77 ± 0.16 ^b^	8.96 ± 0.15 ^b^

Qu, the Huangjiu sample fermented with the original wheat Qu; QT1, the Huangjiu sample fermented with the improved wheat Qu QT1 inoculated with *Lactococcus* T1; QT2, the Huangjiu sample fermented with the improved wheat Qu QT2 inoculated with *Ralstonia* T2; QT3, the Huangjiu sample fermented with the improved wheat Qu QT3 inoculated with *Granulicatella* T3; QT4, the Huangjiu sample fermented with the improved wheat Qu QT4 inoculated with *Acidothermus* T4; significant difference among five samples (*p* < 0.05) was indicated by different letters (a and b).

## Data Availability

The data used to support the findings of this study can be made available by the corresponding author upon request.
